# A Classroom-Based Intervention to Promote Physical Literacy in Children: ALPHYL Study Protocol

**DOI:** 10.3390/bs13070609

**Published:** 2023-07-21

**Authors:** Isaac Estevan, Xavier García-Massó, Cristina Menescardi, Nuria Ortega-Benavent, Sergio Montalt-García, Jorge Romero-Martínez, Isabel Castillo, Octavio Álvarez, Ana Queralt, Javier Molina-García

**Affiliations:** 1 Department of Teaching of Physical Education, Arts and Music, University of Valencia, Avda. dels Tarongers 4, 46022 Valencia, Spain; xavier.garcia@uv.es (X.G.-M.); cristina.menescardi@uv.es (C.M.); nuorbe@alumni.uv.es (N.O.-B.); sergio.montalt@uv.es (S.M.-G.); jorge.romero-martinez@uv.es (J.R.-M.); javier.molina@uv.es (J.M.-G.); 2 AFIPS Research Group, University of Valencia, 46010 Valencia, Spain; isabel.castillo@uv.es (I.C.); octavio.alvarez@uv.es (O.Á.); ana.queralt@uv.es (A.Q.); 3 Department of Social Psychology, Faculty of Psychology and Speech Therapy, University of Valencia, Av. Blasco Ibañez, 21, 46010 Valencia, Spain; 4 Department of Nursing, University of Valencia, Avda. Menendez Pelayo, s/n, 46010 Valencia, Spain; 5 Epidemiology and Environmental Health Joint Research Unit, FISABIO-UJI-UV, 46020 Valencia, Spain

**Keywords:** physically active learning, childhood, physical activity, health, school

## Abstract

Physical literacy is crucial for children’s appropriate development and physical, social and mental health. In a school setting, class-based physical activity (PA) interventions are considered to be appropriate programs to foster PA participation and cognitive development. The purpose of this project, named the Active Learning in PHYsical Literacy (ALPHYL) study, was to describe a multicomponent classroom-based physically-active learning randomised control trial (RCT) in primary school children. The main purpose was to promote children’s physical literacy, academic achievement and cognitive function. The ALPHYL study is mainly based on physical literacy, active school models and the Supportive, Active, Autonomous, Fair and Enjoyable principles. The ALPHYL is an 8–10-week RCT to be conducted in six primary schools (12 classes) in Valencia (Spain) and its metropolitan area. Schools will be randomly assigned to the intervention or waiting-list control group. After a 30 h in-person training course for teachers and weekly meetings in the three months of resource preparation, the ALPHYL intervention will be conducted in physical education (PE) and non-PE lessons by teachers. The intervention consists of at least three daily sessions of physically active learning in addition to model-based PE teaching. Its feasibility will be evaluated weekly according to the Reach, Effectiveness, Adoption, Implementation and Maintenance framework. To assess its effectiveness, a pre-test, post-test and retention (8–10 weeks post-intervention) with primary outcomes (i.e., PA level, motor competence, perceived motor competence and PL, motivation, perceived social support, academic achievement and cognitive function), secondary outcomes and covariates will be collected.

## 1. Introduction

Active play and physical activity (PA) during childhood favours cognitive, social and physical development and affects children’s present and future health [1,2]. Despite its benefits, the percentage of children who comply with the recommendation to perform at least 60 min of moderate-vigorous PA daily is alarmingly low [3,4]. In order to promote compliance with the recommendations, schools have been proposed as ideal settings for promoting active play, PA and reducing sedentary behaviour [5] while stimulating cognitive function [6]. In fact, pupils spend a large proportion of their time (around 7–8 h per day) at school [7]. PA during school hours is associated with aspects such as concentration, cognitive function and academic achievement [8,9,10], even though children remain seated for around 70% of the time in class [8,11,12]. School time could well be the least active and most sedentary period of the day [13]. Given the alarmingly low level of PA practice among young people in developed countries, 30 min of PA at school would contribute to improving their physical, cognitive and mental health [11,14]. If 10 min of sedentary time can be transformed into moderate-vigorous PA (MVPA), positive physical and psychological effects would be evident (e.g., increased motor competence and an improved self-concept) [15]. Implementing interventions that increase school PA levels (e.g., physically active learning) may well be an ideal and low-cost method to improve students’ health and academic achievement [16].

In the educational setting, physical education (PE) can be a favourable context for promoting PA within and outside school hours [5,17]. However, in primary schools, little time is dedicated to PE (7–10% of the weekly timetable) and PE sessions do not always involve MVPA, so the chance to achieve pedagogical goals, such as promoting PA and motor competence, is limited [18,19]. Beyond the PE area, the involvement in subjects like maths, science or languages can provide a significant stimulus to increase the time spent in PA, reduce sedentary time and motivate interest in active lifestyles [5,14]. The implementation of physically active learning, both in PE and other subjects, has the potential to not only improve students’ PA levels during school hours [5], but also increase learning facilitators such as concentration, cognition and time-on-task, as well as executive functions and academic achievement [14]. All of this can be achieved without reducing instructional time in the different areas both in and out of the classroom [11]. To enhance cognitive performance, it seems that more than 20 min daily of MVPA in a physically active classroom is required [20]. So, to improve the PA levels and cognitive function, physically active classroom-based interventions should involve: (a) a length of up to 8–10 weeks and (b) a dosage of about 20–30 min, including 15–20 min of MVPA and another 10–15 min of light PA in which there is mainly cognitive involvement, respectively [11,13].

It is now recognised that the school context is central to the development of students’ physical literacy, which is understood as the motivation, confidence, physical competence, knowledge and understanding of valuing and taking responsibility for performing PA throughout life [21,22,23]. Beyond the mere physical connotation, physical literacy integrates the interrelated physical, psychological, social and cognitive dimensions. It should also be understood as a dynamic and individual construct, which is not acquired on an ad hoc basis, but involves a learning process influenced by the family, school and social environment [21]. Most recent studies have focused on increasing the volume of individuals’ PA, although the impact on qualitative aspects has not received as much attention [24]. It is thus recommended that school interventions should aim at the holistic improvement of students, in addition to maintaining a multicomponent structure (e.g., involving PE and other subjects, families, etc.). In this line, as opposed to approaches whose exclusive aim is to increase the volume of PA, physical literacy seeks to develop knowledge and understanding of how, why and when people move, and the social skills to be active with others [21,24]. However, so far, no multicomponent interventions based on physically active learning have been developed in areas other than PE that would allow us to know whether physically active learning leads to benefits, not only in the volume of PA practice, but also in other health aspects (e.g., enjoyment, social identity, weight status, etc.) and academic achievement. This study thus proposes the development of a multicomponent classroom-based physically active learning randomised control trial (RCT) in primary school children. The main purpose is to empower children’s physical literacy, academic achievement and cognitive function. The physical characteristics of the school and family environments will also be assessed in relation to PA practice and physical literacy during the intervention, which will be based on the theoretical model of creating active schools [14] and physical literacy [21].

## 2. Materials and Methods

### 2.1. Study Design and Participants

The Active Learning in PHYsical Literacy (ALPHYL) study is a RCT in primary schools in Valencia (Spain) and its metropolitan area, with the schools as the units of randomisation and the students as the units of analysis. The design, implementation and reporting of ALPHYL will be carried out according to the CONSORT statement [25] and considering the SPIRIT recommendations. The sample will consist of at least 264 primary school Grade 5 pupils (i.e., 10–11 years old).

The sample size was calculated from previous studies that focused on the effects of interventions on PA and motor competence [11,22], using G*Power 3.1 software (University of Düsseldorf, Düsseldorf, Germany). From these studies, for an effect size between 0.30 and 0.32 with an alpha level of 0.05 and statistical power of 90%, the sample size had to be between 105 and 220 participants. To avoid reducing statistical power due to experimental mortality, the sample was increased by 20%, with the participation of 264 children. To recruit the participants, children from six schools (three intervention and three control schools; four public and two private) in Valencia and its metropolitan area will take part, consisting of pupils willing to participate and whose families provide signed informed consent (inclusion criterion). Pupils who present evident physical and mental difficulties that prevent them from satisfactorily completing all the measurements proposed in the study will not be included in the analyses (exclusion criteria). Only typically developing children will be included in the analysis.

### 2.2. Patient and Public Involvement

Students were not involved in the design of this study. Students and teachers will be involved in the conduct, reporting and dissemination plans of this research. The teachers of the schools involved in this study did participate in the design of this study. Moreover, we will continue to involve these teachers as we complete the study, through regular meetings to obtain their feedback on the study.

### 2.3. Procedure

**Figure 1:**
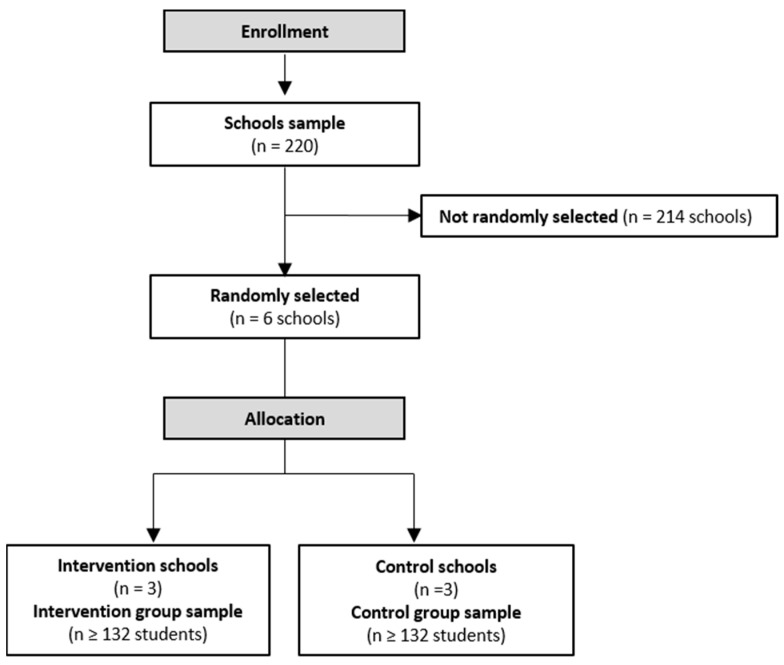
Plan flow chart of the ALPHYL study.

The CONSORT study flow diagram is shown in Figure 1. A list of primary schools in Valencia and its metropolitan area will be obtained from the government educational institutions. The schools will be randomly selected and ordered in a list using computer-generated random numbers by a researcher from the study team. The inclusion criteria for the schools’ participations will be: (a) must have at least two groups of primary school Grade 5 pupils, and the teachers involved in both must agree to participate; (b) each class group includes at least 75% of the participants; and (c) children must not have taken part in other PA promotion interventions during the intervention period in the previous two years. If a school decides not to participate, the same procedure will be performed with the next school selected from the list. The six selected schools will also be randomly allocated to the intervention (*n* = 3) or control groups (*n* = 3). Students will be unaware of whether they have been allocated to the intervention or control group.

In order to detect barriers to and facilitators of the intervention’s adoption, implementation and sustainability, an instrument based on the reach, effectiveness, adoption, implementation and maintenance (RE-AIM) tool will be used to analyse its reach, effectiveness, adoption, implementation and maintenance [26], as well as its impact [8,26]. This instrument will be implemented with the teachers involved in the intervention, who will provide information on their teaching methods and the pupils’ participation by completing a personal teaching diary [13,27].

### 2.4. Intervention

The intervention will last for approximately 8–10 weeks. Systematic review evidence by Norris et al. [11] indicated that if the duration lasts longer than 8 weeks and the total volume in minutes is less than those proposed in the current study, the acute effects on some of the main variables (e.g., PA or academic achievement) could be reduced. Another recent school-based intervention [28] conducted over 8 weeks found positive effects on motor competence. To ensure that all the groups receive a similar amount of physically active learning, three research assistants with expertise in PE and classroom-based physically active learning will accompany the teaching team during the entire intervention. Their role will be to support, assist and collaborate in the development of resources, monitor their implementation, and ensure that the lessons are delivered according to the study’s guidelines.

In addition to the PE lessons, at least three physically active activities in subjects other than PE will be conducted every day (5 days per week), with a total duration of approximately 30 min of light and MVPA per day. The weekly dose of physically active learning will thus be 100–150 min, so that a volume of 800–1200 min is foreseen during the 8–10 weeks. All three of the physically active lessons per day consisted of transforming and delivering traditional teaching in maths, science, social science, language, etc., by including a movement-based activity in the work program. No instant restriction will be used for physically active learning. For instance, during a typical lesson of science wherein pupils are sitting in their chairs, the classroom teacher and the research assistant will propose them to move to the corridor, hall or any available open space to conduct the physically active activity. Conversely, in the control schools, the whole teaching process will be conducted in a regular manner following the formal curricula, which traditionally involves having the pupils sit for the whole lesson.

The PE classes at both the intervention and control schools will also be conducted following the formal curricula. On the one hand, at intervention schools, PE will be based on a hybridisation of cooperative learning, non-linear pedagogy and health-based PE [29,30,31,32], which are in accordance with the current law of education. On the other hand, at control schools, PE teachers will deliver their own traditional program that is also based on the law of education (i.e., personal knowledge and autonomy, fundamental movement skills, corporal expression, physical activity and health, and sport and games).

The promotion of physical literacy will be based on increasing motor competence, as well as self-perception and motivation for PA, positive affective within the group (e.g., enjoyment and fun), interdisciplinary knowledge (i.e., specific content of physically active lessons and PE) and a sense of personal and social responsibility. To foster healthy interpersonal skills, teaching quality will be enhanced by a coherent sense of identity, the ability to act as a responsible, moral and social member of the group [33], and the learners’ holistic development. Supportive, Active, Autonomous, Fair and Enjoyable (SAAFE) principles [34] will be the basis of the intervention.

To encourage family support for PA practice and a healthy lifestyle, three reports will be sent to the families during the intervention to share behavioural change recommendations for the following: first, playing with the children using novel easy-to-construct materials; second, assessing psychosocial support for the children; and third, actively travelling to the school. From the third week of intervention, teachers will share each report with the families by using the social media (i.e., Telegram) they commonly use to communicate with them 15 days apart. The reports will be available on the ALPHYL webpage.

Table 1 shows the intervention program. The classroom teachers involved will be accompanied on two fronts: by the school’s PE teachers and a research assistant who will act as a guide and/or reference to organise physically active learning. On the other hand, the co-created resources (i.e., cards, sheets or any required resource in the physically active learning activities) will be made available to them. PE and other teachers will receive training and support for the implementation of the trial. They will be provided with the proposals that will be explained, put into practice and evaluated during the training course. Before and after the trial, two semi-structured focus-group meetings will be conducted with the teachers involved.

### 2.5. Measurements

Table 2 describes the different primary and secondary outcomes that will be assessed. These indicators are classified into individual factors according to the different physical literacy domains and the environment created. All the measurements will be carried out during school hours in two PE lessons and two regular classroom lessons in each group for pre-test, post-test and retention.

During PE lessons, three measures will be performed: (1) Actual motor competence by using the Canadian Agility Movement Skill Assessment (CAMSA), a circuit-based field-test test [35,36] which combines product- and process-oriented fundamental movement skills measures. This test is feasible in PE classes [36]. (2) The Progressive Aerobic Cardiovascular Endurance Run (PACER) will also be used to assess cardiorespiratory fitness [50], as well as, (3) children’s weight and height. Barefoot weight and height will be measured once with participants wearing light clothes. The body mass index (BMI) will be calculated as weight in kilograms divided by height in square metres (kg/m^2^) and determined according to the children’s sex and age [49].

The pupils will be given a questionnaire in a classroom setting with different measures, including perceived motor competence [39,40] and physical literacy [38], motivation [41,42], perceived social support [43,44], active commuting to school [48], PA participation [42,51], alienation with school [52], future PA intention [43], psychological needs satisfaction [41], perceived body image [53], physical self-concept [54], sedentary behaviour [56], school satisfaction [57], social identity [58], knowledge related to PA and healthy lifestyles [41,59] and the measurement of math fluency calculation [60]. Two cognitive function tests, Stroop and digit-span tests [47], will be carried out individually, while academic achievement will be obtained from the final grade scores [45,46].

After these measurement sessions, each participant will be given an ActiGraph wGT3X-BT accelerometer (ActiGraph Corp ^®^, Pensacola, FL, USA, EEUU) to wear on a hip belt for 8 days [15], a method that has shown validity and reliability in assessing PA patterns in children [37].

#### Covariates

In addition to children’s sex and age, parental educational qualifications will be assessed to estimate the families’ socioeconomic status (SES) using a parental questionnaire. This will be assessed by the mother and father’s reported qualifications, something that was completed by any elementary, middle, high school or university [61].

The measurements of the perception of the characteristics of the participants’ home neighbourhoods will be carried out on the Neighbourhood Environment Walkability Scale for Youth (NEWS-Y-IPEN) questionnaire [62], which will be filled out by the parents. The NEWS-Y-IPEN provides information on the neighbourhood characteristics regarding aspects such as traffic safety, infrastructures, diversity of destinations, etc. (Table 3). The families will indicate their postal address to enquire into the objective characteristics of the home neighbourhood. Geographic information systems (GIS) and systematic observation tools will be used to analyse the environments, including the connectivity of the streets or the presence of the green areas that young people have at their disposal to maintain active behaviour. The physical school characteristics will be assessed in relation to PA [63,64] using an audit process. The schools’ neighbourhood environment will also be evaluated by GIS procedures and the Microscale Audit of Pedestrian Streetscapes (MAPS) Global audit tool [65].

### 2.6. Data Management and Statistical Methods

To avoid possible bias in the data analysis, an independent blinded researcher will carry out a pseudonymisation process to guarantee the anonymity of the data. Missing data will be imputed using multiple imputation. According to our Institutional Review Board (IRB), a data monitoring committee will not be necessary since this is a low-risk study. Adverse events and protocol deviation will be documented by teachers using their personal teaching diary. Moreover, the research team will perform weekly audits to ensure data quality and the correct development of the protocol.

Independent researchers will also code actual motor competence videotapes so that they are stratified and counterbalanced. The video coded data, information from the individual questionnaires, accelerometer data and a family proxy-report of the participants’ residential neighbourhood will be hosted on an open access digital platform (zenodo.org) to be available to the scientific community.

The teachers’ data obtained in the semi-structured interviews will be categorised and thematically analysed by the NVivo^®^ qualitative analysis program (QSR International: Technology & Software Solutions, Burlington, MA, USA).

Structural equation modelling will be conducted to analyse the association and mediating role of some of the measured variables according to the conceptual models of children’s motor development and physical literacy [21,66]. To study the effect of the intervention on primary and secondary variables, linear mixed models will be built with the time, condition and interaction of time and condition as fixed effects, adjusting for baseline values. When a significant interaction effect is found, post-hoc tests will be applied by the least significant difference method. The data will be analysed on different statistical software, mainly SPSS (SPSS Inc., Chicago, IL, USA) and Mplus [67].

An analysis of the intervention effects in a person-centred approach will allow us to determine the children’s profiles according to their primary physical literacy outcomes (i.e., actual and perceived motor competence, motivation, perceived social support and cognitive function), while the trends and association will be analysed with the remaining physical literacy primary and secondary outcomes, for which self-organised maps will be created on MATLAB (MathWorks Inc., Natick, MA, USA).

## 3. Strengths and Limitations

The strengths of the ALPHYL study include the study design (i.e., randomised controlled trial), the use of a comprehensive set of individual, social and environmental assessments to evaluate the effects of physically active learning on physical literacy, and the assessment of the implementation process guided by the RE-AIM framework and SAAFE principles. A theory-based procedure (i.e., Active Schools, Physical Literacy and Health-based Physical Education models) designed to implement the intervention is another study strength. The main limitation of this study protocol is the lack of assessment of children’s maturity at baseline and post-intervention.

## 4. Ethics and Dissemination

The study was approved by the Ethics Committee of the University of Valencia (reference: UV1259844). The findings of the ALPHYL study will be disseminated in the academic community via publications and conferences. The results will be shared with key education stakeholders and policymakers with a set of guidelines to implement future classroom- and school-based PA interventions with the aim of fostering physical literacy among children. The findings will also provide valuable and comprehensive information for the participating schools on their pupils’ physical literacy and its relationship with outcomes such as cognitive function and academic achievement. This trial has been registered at Clinicaltrials.gov, number NCT05812118.

## Figures and Tables

**Table 1:** Study phases and intervention structure.

Components	Date	Components Aim	Assessment
General teachers training (15 h)	May–July 2022	To empower PE and classroom teachers in the acquisition of knowledge, competence and abilities in conducting the ALPHYL intervention.	Focus group (first round) with PE and classroom teachers.
Specific by subject (9 h)			
Applied sessions (6 h)			
Resources co-creation (building & application)	September–December 2022	To support and promote teachers’ self-confidence in creating ecological resources to their own school setting in correspondence with the ALPHYL intervention (e.g., distribution of spaces, groups management, realistic physically active activities, etc.).	-
Teachers advising			
Pre-test. Primary/secondary outcomes	January 2023	To analyse participants’ socio-demographics, physical literacy and built environment of schools and residencies before the intervention.	Primary outcomes, secondary outcomes and covariates.
Covariates			
RCT. ALPHYL intervention	January–March 2023	To deliver the ALPHYL intervention by PE and classroom teachers who will be accompanied and supported by a research assistant with knowledge, competence and ability conducting physically active learning.	Primary outcomes, secondary outcomes and fidelity.
Post-test	March 2023	To analyse participants’ physical literacy immediately after the intervention.	Primary and secondary outcomes. Focus group (second round) with PE and classroom teachers.
Retention	May–July 2023	To analyse participants’ physical literacy 8–10 weeks after the intervention.	Primary and secondary outcomes.

**Table 2:** Primary and secondary outcomes of the study.

Variable	Instrument/Description	Key Reference/s
**Primary physical outcomes**		
Actual MC	Canadian Agility Movement Skill Assessment test (CAMSA)	[35,36]
PA level	ActiGraph wGT3X-BT (ActiGraph Corp, Pensacola, FL, USA, EEUU).	[37]
**Primary psychological outcomes**		
Perceived MC and physical literacy	Pictorial scale of Perceived Movement Skill Competence (PMSC) and Physical Literacy for Children Questionnaire (PL-C Quest)	[38,39,40]
Self-determined motivation for PA	Adapted version for children of the Behavioural Regulation Exercise Questionnaire	[41,42]
**Primary social outcomes**		
Perceived social support	The Physical Activity Family and Friends Support Scale (PASS)	[43,44]
**Primary cognitive outcomes**		
Academic achievement	Final grade scores	[45,46]
Cognitive function	Stroop and Digit Span test	[47]
**Secondary physical outcomes**		
Active commuting to/from school	Survey adapted from Centers for Disease Control Kids-Walk-to-School program by IPEN investigators	[48]
Body Mass Index	Height (SECA standardised stadiometer), weight (TANITA, BC-601; Tanita Corporation of America, Inc, Arlington Heights, IL, USA)	[49]
Cardiorespiratory fitness	Progressive Aerobic Cardiovascular Endurance Run (PACER)	[50]
PA participation (Self-reported)	Physical Activity Questionnaire for Children (PAQ-C)	[42,51]
**Secondary psychological outcomes**		
Alienation with school	Health Behaviour in school-age children	[52]
Body image perception	Children Body Figure Silhouette scale	[53]
PA intention	Future intention of PA scale	[43]
PA psychological need satisfaction	Basic psychological needs satisfaction within a PE setting	[41]
Physical self-concept	The pictorial scale of Physical Self-Concept in Children (P-PSC-C)	[54,55]
Sedentary behaviour	Youth Leisure-Time Sedentary Behaviour Questionnaire (YLSBQ)	[56]
**Secondary social outcomes**		
School satisfaction	Intrinsic Satisfaction Classroom Scale	[57]
Social identity	Social Identity Questionnaire for Physical Education/Sport (SIQS)	[58]
**Secondary cognitive outcomes**		
Knowledge related to PA and healthy lifestyles	Self-Determination index	[41,59]
Math fluency calculation	The Spanish version of the Woodcock-Johnson III (6th test)	[60]

Note. PA, physical activity. MC, motor competence.

**Table 3:** Study covariates.

Variable	Instrument/Description	Key Reference/s
Socio-demographics (children’s sex and age, and parental educational level)	Parental questionnaire	[62]
Physical and built environment		
School and home neighbourhood built environments	GIS-based data (ArcGIS 10.2 software; ESRI, Redlands, CA, USA, EEUU) and MAPS-Global audit tool	[48,65]
Internal physical school characteristics in relation to PA	International Study of Childhood Obesity, Lifestyle and the Environment (ISCOLE) school audit tool (ISAT)	[63,64]
Parental perceptions of the home neighbourhood built environment	NEWS-Y (Neighbourhood Environment Walkability Scale for Youth) IPEN	[62]

Note. PA, physical activity.

## Data Availability

Data from this project will be available in zenodo.org.

## Abbreviations

ALPHYL	Active Learning in PHYsical Literacy
BMI	Body Mass Index
CAMSA	Canadian Agility Movement Skill Assessment
GIS	Geographic Information Systems
IRB	Institutional Review Board
MAPS	Microscale Audit of Pedestrian Streetscapes
MVPA	Moderate-Vigorous Physical Activity
NEWS-Y	Neighbourhood Environment Walkability Scale for Youth
PA	Physical Activity
PACER	Progressive Aerobic Cardiovascular Endurance Run
PE	Physical Education
RCT	Randomised Control Trial
RE-AIM	Reach, Effectiveness, Adoption, Implementation and Maintenance
SAAFE	Supportive, Active, Autonomous, Fair and Enjoyable
SES	Socioeconomic Status
